# SNP-SIG Meeting 2011: Identification and annotation of SNPs in the context of structure, function, and disease

**DOI:** 10.1186/1471-2164-13-S4-S1

**Published:** 2012-06-18

**Authors:** Yana Bromberg, Emidio Capriotti

**Affiliations:** 1 Department of Biochemistry and Microbiology, School of Environmental and Biological Sciences, Rutgers University, New Brunswick 76 Lipman Drive, NJ 08901, USA; 2 Department of Mathematics and Computer Science, University of Balearic Islands, Ctra. de Valldemossa Km 7.5, Palma de Mallorca, 07122 Spain

## Overview

Advances in high-throughput sequencing, genotyping, and characterization of haplotype diversity are consistently generating vast amounts of genomic data. Single Nucleotide Polymorphisms (SNPs) are the most common type of genetic variation. In the recent years the number of known SNPs has been increasing exponentially; the last release of the NCBI’s dbSNP database contained more than 50 million human SNPs. SNPs are interesting as both markers of evolutionary history and in the context of their phenotypic manifestations (*e.g.* characteristic traits and diseases). For some diseases, *e.g.* sickle-cell anemia, the causative SNPs are well documented. In most other cases, the detection of disease-causing variants is still a problem. The genome-wide association studies (GWAS) provide insight into SNP-disease relationships. However, GWAS analysis is both experimentally and computationally expensive and fails to properly consider the rare variants, *i.e.* individual-specific SNPs that have yet to be documented on a population scale. This discrepancy between the deluge of SNP data and the lack of its interpretation spurs the development of the SNP impact annotation/prediction algorithms. In the near future, the study of genetic variation in disease and treatment options will be key for the development of the field of personalized medicine.

In 2010, the first edition of the Critical Assessment of Genomic Interpretation (CAGI; Berkeley, California) was organized to evaluate the ability of available computational methods to predict the phenotypic impacts of genomic variation. Annotation of SNPs was also a hot topic in many other meetings, such as AIMM at ECCB 2010 (Ghent, Belgium), the HGVS 2010 meeting (Washington, DC) and PSB 2011 (Big Island of Hawaii, USA). In line with the increasing interest in the genetic variation analysis and annotation, on July 15^th^, 2011 we organized the first SNP Special Interesting Group (SNP-SIG) meeting at ISMB/ECCB’2011 in Vienna, Austria (http://snps.uib.es/snp-sig/2011). This meeting attempted to summarize the field’s research advances in the directions of “Annotation and prediction of structural/functional impacts of coding SNPs” and “SNPs and Personal Genomics: GWAS, populations and phylogenetic analysis”. Over 70 scientists actively working in the field and strongly interested in its development have officially registered for the SIG. On the date of the meeting, an even larger number of ISMB participants have gathered to discuss their work, the state of the art, and future perspectives. In all, 17 presentation proposals and 13 posters were submitted to the SIG and eight works where selected for an oral presentation at the meeting.

Distinguished scientists were invited to share their visions of the field past, present, and future: Steven Brenner (University of California at Berkeley), Atul Butte (Stanford University), John Moult (University of Maryland, College Park), Burkhard Rost (Techinal University of Munich) and Mauno Vihinen (Lund University). A round table discussion on the most timely and important problems of SNP annotation was held, directed by Christopher Baker (University of New Brunswick), Maricel Kann (University of Maryland, Baltimore), Sean Mooney (Buck Institute), Pauline Ng (Genome Institute of Singapore) and Mauno Vihinen (Lund University).

We have invited all SIG presenters to submit full research papers for publication in this special issue of BMC Genomics. We adopted a peer review process to select ten exceptional works. The articles cover different aspects of the field, presenting databases and tools for the annotation of SNPs as well as novel scientific advances achieved based on these resources. The described methods use different types of information derived from sequence, evolution, function and structure to analyze large sets of variations. They address SNP-associated (1) specific protein function [1] or (2) structure/stability [2,3] changes or focus on (3) non-coding SNPs [4,5] or (4) specific disease classes [6,7]. Presented work gives new life to the information buried in literary free text [8] and outlines the potential of using SNP functional impacts to predict disease involvement [9]. We also include a *method developer* tutorial/framework that will be very helpful for all future work in the field [10].

## Next meeting

We are now working on the organization of the next edition of the SNP-SIG meeting to be held in the context of the ISMB 2012, Long Beach, California. Further information about the SNP-SIG 2012 is available on our web site (http://snps.uib.es/snp-sig). Submissions of posters and presentation proposals are welcome.
